# Efficacy of bifidobacterium-related preparations on depression: the first meta-analysis

**DOI:** 10.3389/fpsyt.2024.1463848

**Published:** 2024-10-03

**Authors:** Ruihan Huang, Yongsheng Liu

**Affiliations:** ^1^ School of Public Health, Qilu Medical University, Zibo, Shandong, China; ^2^ School of Clinical Medicine, Qilu Medical University, Zibo, Shandong, China

**Keywords:** psychobiotics, bifidobacterium, depression, treatment outcome, meta-analysis

## Abstract

Currently, depression-induced suicide has emerged as the primary contributor to the worldwide burden of disability. However, the prevailing drug treatment not only suffers from delayed effectiveness and limited efficacy, but also there are withdrawal symptoms and rebound phenomenon. Consequently, there is an imperative to investigate safer and more efficient treatments to ameliorate the clinical manifestations of depression. At present, there is increased evidence that probiotics can improve the symptoms of depression, but the existing studies use many and mixed types of probiotics, and it is impossible to determine the specific efficacy of bifidobacteria in the treatment of depression. This review will systematically review the effects of bifidobacteria on the treatment effect of depression, Meta-analysis showed that Bifidobacterium-related preparations effectively improved depressive symptoms in patients with depression. This study represents the initial meta-analysis conducted on the use of bifidobacteria-related agents for treating depression. The objective was to determine the effect of bifidobacteria-related preparations on improving depressive symptoms. We found that Bifidobacterium and its related agents can effectively reduce depression scale scores in patients with depression, suggesting the need for further research into this potential strategy for the prevention and treatment of depression.

## 1 Introduction

Depression is a prevalent psychiatric condition that can impact individuals indiscriminately. It is characterized by a prolonged and intense state of sadness, and some individuals may exhibit self-injurious behavior, suicidal tendencies, and in severe cases, may experience psychotic symptoms like delusions and hallucinations. According to the latest statistics of the World Health Organization (WHO), there are about 280 million people with depression in the world, accounting for about 3.8% of the total population, and the incidence rate of women is about 50% higher than that of men. Worldwide, more than 10% of pregnant women and women who have just given birth suffer from depression, and the lifetime prevalence of major depressive disorder ranges from 3% in Japan to 16.9% in the United States, resulting in suicide as the fourth leading cause of death among people aged 15-29 years (60).

Depression brings a heavy economic burden to the family and society (1), and the current treatment of depression is mainly based on medication, but more than half of patients with major depressive disorder still have insufficient response to antidepressants (2–4, 7) and in special populations such as children, adolescents, and pregnant women, the availability of therapeutic agents is relatively limited (8). Therefore, further exploration of safer and more effective drugs remains an urgent need (5, 6). Existing research suggests that probiotics may be able to play a significant role in the prevention and treatment of depression and have the advantage of being less addictive and less side effects (9).

The basis for the mechanism of action of probiotics on depression stems from the interaction between the brain and gut microbiota. Intestines. The emerging concept of the brain axis suggests that the gut microbiota is closely related to mental disorders such as depression (10). Recent research has also shown that Probiotics can not only protect intestinal integrity but also enhance intestinal function. It can also affect brain function (11). This, in turn, enhances the survival and differentiation of neurons. Probiotics have been shown to be an adjunct therapy for the treatment of metabolic and psychiatric disorders (12, 13)and have been shown to be a variety of urgent, adjunctive therapeutics for chronic infections, cancers, inflammatory diseases, and cognitive and psychiatric disorders (14, 15). It is expected to become an alternative to various chemicals, synthetic drugs and antibiotics (16). The regulation of gut microbiota by probiotics can alter brain function through the gut-brain axis and regulate mood (17, 18), which can have health benefits for people with mental illness when ingested in sufficient amounts. These probiotics are called psychoprobiotics (19), which communicate with the brain mainly through the pathway of vagus nerve, tryptophan metabolites and microbial products (20), By producing and delivering neuroactive substances that directly or indirectly regulate cognitive and emotional states (21).

Recent meta-analyses have confirmed the effectiveness of probiotics in treating depression. A meta-analysis of 13 randomized controlled trials supported the use of probiotics in people with mild to moderate depression (22). The results of another meta-analysis of 34 controlled clinical trials support the efficacy of probiotics for depression and anxiety, and probiotics were observed to have the greatest effect on major depressive disorder (6). However, the heterogeneity of these studies in terms of probiotic strains makes it impossible to determine the different effects of different strains on improving depressive symptoms. Therefore, our study will narrow down the scope of probiotics and conduct a meta-analysis of controlled clinical trials of the efficacy of Bifidobacteria and their related agents in the treatment of depression.

Bifidobacterium is a typical intestinal probiotic, it is a gram-positive, non-motile, non-spore-forming obligate anaerobe, widely found in the digestive tract, vagina and oral cavity of humans and animals, and is one of the earliest and most abundant bacterial colonizers of the neonatal intestine (23), which accounts for more than 90% of the total microbiota in the infant’s gastrointestinal tract (24). Bifidobacteria are present in the human gastrointestinal tract since birth and throughout human life. Studies have shown that several diseases are associated with decreased levels of bifidobacteria in the human gut microbiota (25, 26). Therefore, the content of bifidobacteria in the intestinal flora is of great significance for maintaining human health (27).

Bifidobacterium is one of the most widely used probiotics (28), which can have a positive impact on the intestine by reshaping the microecological balance inside the intestine (29) and improve the intestinal flora disorder in patients with depression. Studies have shown that long-term use of bifidobacterium can produce similar effects to antidepressants on relevant brain regions (30, 31). There is growing evidence that bifidobacteria can improve symptoms of depression.

## 2 Methods

### 2.1 Inclusion criteria

According to the PICOS principles, the implementation is as follows: (1) the study subjects are patients with clear depressive symptom definition criteria, the course of the disease is not limited, the age is ≥ 18 years old, and voluntary participation; (2) The control group received conventional treatment or placebo; (3) Outcome measures: Hamilton depression, ruminant thinking, quality of life, four scales of Pittsburgh Sleep Quality Index, self-compassion questionnaire, mindfulness factor level, suicidal ideation; (4) The literature type was randomized controlled trials (RCTs) in Chinese and English.

### 2.2 Exclusion criteria

Exclusion criteria: (1) non-RCTs versus conference articles; (2) animal experiments, case studies, meta-analyses, and reviews; (3) The data is incomplete, and the full text cannot be obtained; (4)Tients with mental disorders other than depression.

### 2.3 Search strategy

Two reviewers used the keywords [bifidobacterium] and [depression OR depressive symptoms OR depressive symptom OR symptom, depressive OR emotional depression OR depression, emotional] in PubMed, EMBASE, Cochrane Library, China Journal Full-text Database, Wanfang Database, and China Biomedical Literature Database were searched and screened. The formulation of literature search strategy adopts techniques like combining subject headings and free words, restriction fields and Boolean logic. References and citations of RCTs and related reviews were also traced back to further ensure the recall of the literature search. Each search result was independently reviewed by two independent researchers, regardless of language, and the search time was from inception to March 2024.The search process is to first remove the duplicate literature, and then conduct a preliminary screening according to the title and abstract of the literature, determine the literature that needs to be screened for secondary screening, and obtain the full text. Read the full text to select eligible studies, i.e., the RCTs for inclusion. If data from eligible RCTs are incomplete, we will contact the corresponding authors by email to obtain the full data.

### 2.4 Data collection

The following data were extracted from the included RCTs: 1) first author, year of publication, country, title, etc.; 2) the diagnostic (screening) criteria, sample size, age, gender, interventions, comparators, course of treatment, and follow-up period of the study subjects; and 3) primary outcome measures and corresponding data (including after intervention and long-term follow-up).

### 2.5 Literature quality assessment and statistical analysis

Two authors independently assessed the quality of the included studies using the RCT risk of bias assessment tool from the Cochrane Handbook for Systematic Review of Interventions. Analyses using RevMan version 5.3 were used to provide 95% confidence intervals (CIs) for the effect of quantitative data, depending on whether the same measurement method and unit was used, weighted mean difference (WMD) or standardized mean difference (SMD). The I2 statistic and Q statistic were used to test for heterogeneity, and if I2 ≥ 50%, the results were considered to have significant heterogeneity, and the random-effects model was used to calculate the parameters. Conversely, if heterogeneity was small, a fixed-effect model was used for meta-analysis, P < 0.05 was statistically significant. Funnel plot tests were used to assess publication bias. Sensitivity analyses were performed by excluding studies on a case-by-case basis. We also performed subgroup analyses by geography and type of depression. We analyzed the risk of bias for the efficacy of probiotics for each of the included randomized controlled trials using the Cochrane risk assessment tool. If there is significant clinical heterogeneity, subgroup analyses can be used to investigate the source of heterogeneity. Sensitivity analysis was performed using the case-by-case elimination method, and the funnel plot and Egger test were used to analyze the publication bias.

## 3 Results

### 3.1 Included studies

**Figure 1:**
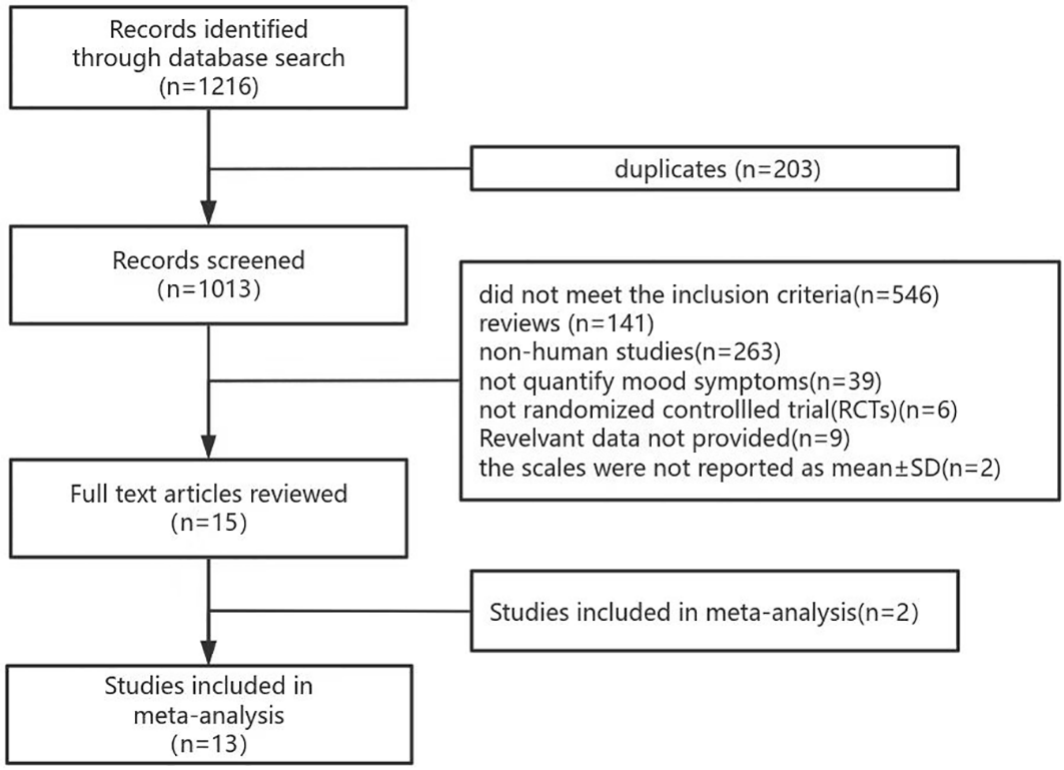
PRISMA flow chart of literature search.

1216 records were retrieved from the six databases and 203 duplicate records were deleted. After screening titles, abstracts, and full texts, 13 studies were finally included. A detailed screening flowchart for meta-analysis is shown in **Figure 1**.

### 3.2 Basic characteristics of the included studies

The 13 randomized controlled trials (RCTs) published between 2015 and 2024 involved individuals with depressive symptoms recruited from nine different countries. Out of these RCTs, three included patients diagnosed with major depressive disorder. The intervention measures were Bifidobacterium and its related preparations, and the intervention form included 6 lyophilized powders, 2 Bifidobacterium quadruple viable tablets, 4 capsules, and 1 spray-dried powder, and the course of treatment was 4 weeks ~ 12 weeks. Outcome measures involved scores on four self-rating scales for depressive symptoms, and in all cases, higher scores were associated with more severe depressive symptoms. See **Table 1** for details.

**Table 1:** Characteristics of included RCTs for meta-analysis.

Author, year	Study sample	Duration	Bifidobacterium -related preparations	Clinical Measure	Country of origin
Akkasheh, 2016 (32)	N=40, MDD patients, Age 20 to 55 years	8 weeks	Capsule, 2×109 CFU	BDI	Iran
Boehme, 2023 (33)	N=45, healthy adults, Age 25 to 65 years	6 weeks	Dry powder, 1×1010 CFU	HADS-D	Switzerland
Chen, 2022 (30)	N=80, patients with PSD, Age 44 to 68 years	8 weeks	Bibiotic tetrad tablets,0.5×107 CFU,3 times/day	HAMD	China
Gawlik-Kotelnicka, 2023 (34)	N=44, patients with Dep, Age >18 years	60 days	Capsule, 3×109 CFU	MADRS	Poland
Haghighat, 2021 (35)	N=65, patients with HD,Age 30 to 65 years	12 weeks	Freeze-dried,2.7×107 CFU	BDI	Iran
Kazemi, 2019 (36)	N=54, Clinical MDD patients, Age 18 to 50 years	8 weeks	Freeze-dried,10×109 CFU	BDI	Iran
Lee, 2021 (37)	N=156, healthy adults, Age 19 to 65 years	8 weeks	Capsule,2.5 × 109CFU	BDI	Korea
Patterson, 2024 (38)	N=83, healthy adults, Age 18 to 45 years	8 weeks	Capsule,1×109 CFU	HADS-D	Irdland
Pinto-Sanchez, 2017 (39)	N=44, patients with IBS and mild to moderate anxiety and/or Dep, Age 26 to 58 years	10 weeks	spray-dried,1 × 1010 CFU	HADS-D	Canada
Romijn, 2017 (40)	N=79, patients with Dep, Age>16 years	8 weeks	Freeze-dried, ≧̸3×109 CFU	MADRS	New Zealand
Steenbergen, 2015 (41)	N=20, healthy adults, Mean age 20.2years ± 2.4	4 weeks	Freeze-dried,>2.5×109 CFU	BDI	Netherlands
Tian, 2022 (42)	N=45, patients with MDD,Mean age 51.32 years ± 16.11	4 weeks	Freeze-dried,1010 CFU	HDRS-D	China
Yao, 2023 (43)	N=108, patients with Dep, Age 49 to 74 years	12 weeks	Bibiotic tetrad tablets,0.5×107 CFU 3 times/day	HAMD	China

MDD, Major depressive disorder; Dep, Depression; IBS, Irritable bowel syndrome; BDI, Beck Depression Inventory; HADS-D, Hospital Anxiety and Depression Scale; HAMD, Hamilton Depression Rating Scale; MADRS, Montgomery Asberg Depression Rating Scale.

### 3.3 Quality assessment

**Figure 2:**
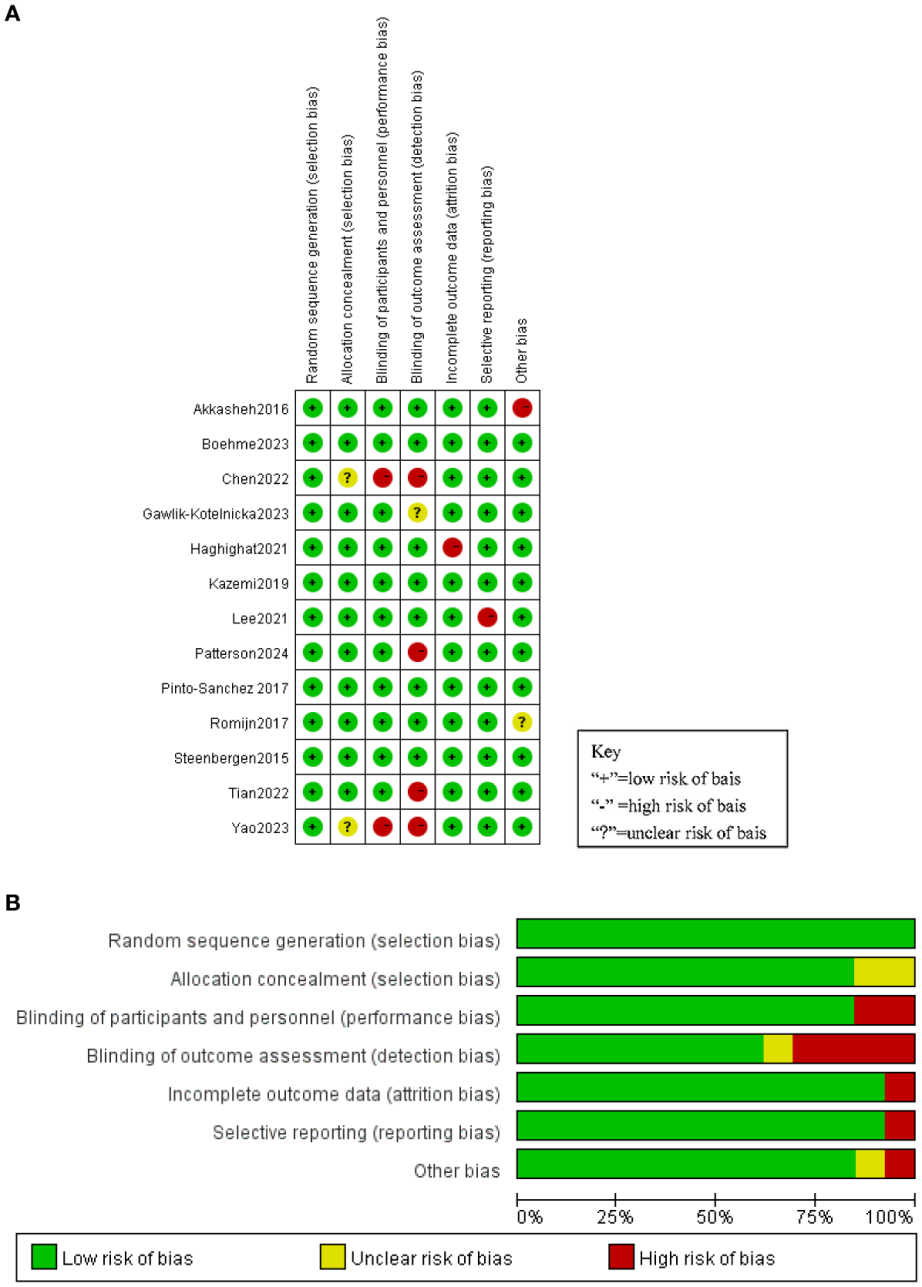
**(A)** Risk of bias summary. **(B)** Risk of bias graph.

The interventional studies included in the review were evaluated based on the Cochrane risk assessment tool. Of the 13 intervention studies, four were classified as ‘low risk of bias’, two as ‘some problems’ and seven as ‘high risk’. The risk of bias of all studies is shown in **Figure 2**.

**Figure 3:**
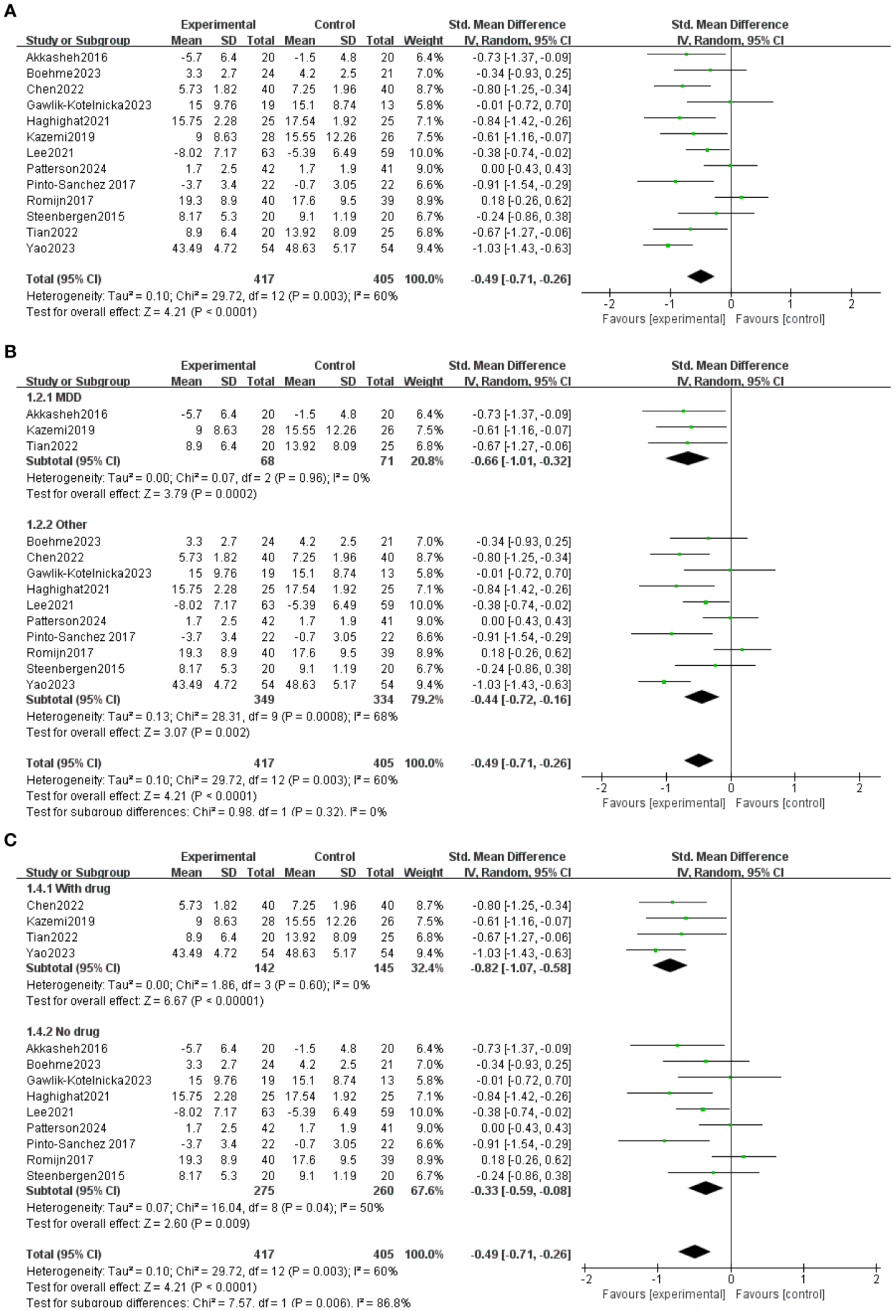
**(A)** General forest map of bifidobacteria and their related agents for the treatment of depression. **(B)** Forest plot divided into two subgroups, “major depressive disorder” and “other”, according to the severity of depressive symptoms. **(C)** Forest plots divided into two subgroups: “taking drugs” and “not taking drugs” according to whether they took other drugs.

All 13 trials included in the study used probiotic preparations that contained Bifidobacterium. The duration of probiotic administration in each trial varied from 4 to 12 weeks. All 13 studies reported baseline data for each group. There were no statistically significant differences between baseline data within and between groups in the Bifidobacterium and placebo groups. At the end of treatment, Depression was lower in the 11-event bifidobacteria group than in the placebo group (30, 43, 32–37, 39, 41, 44). No significant difference was observed between the bifidobacterial group and the placebo group in two items (38, 40). As shown in **Figure 3A**, Meta-analysis comparing Bifidobacterium with controls showed MD= -0.49 (95%CI:-0.71, -0.26), p<0.0001.

The participants were divided into two groups for subgroup analysis based on their use of other medications. This division allowed us to observe the effects of the combination of bifidobacteria and the depression scale (**Figure 3B**) in a more focused manner. Four of the studies took other antidepressants with MD = -0.82 (95% CI: -1.07, -0.).58), p<0.00001; Nine studies took only Bifidobacteria, MD = -0.33 (95% CI: -0.).59, 0.08), p = 0.008. Subgroup analyses were performed according to the type of depression, and all studies were classified as major depressive disorder and others (**Figure 3C**). Three studies were for major depressive disorder, MD = -0.66 (95% CI: -1.01, -0.32), p = 0.0002; Ten studies included six studies with mild to moderate depression and four healthy volunteers with subclinical depressive symptoms MD = -044(95%CI:-0.72, -0.16), p=0.002.

### 3.4 Publication bias

**Figure 4:**
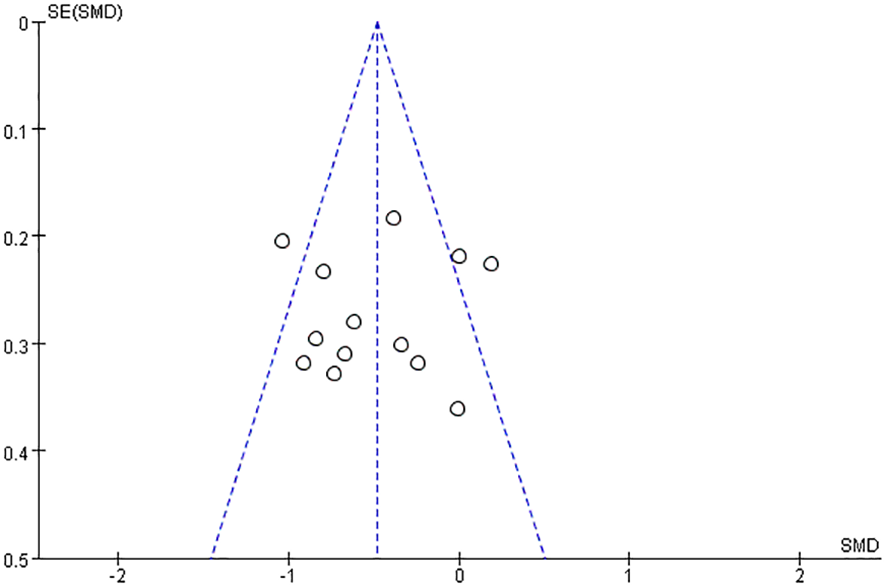
Funnel plot of bifidobacteria and their related agents for the treatment of depression.

Regarding publication bias, the funnel plot is shown in **Figure 4** and no significant asymmetry was observed in the funnel plot, so there was no evidence of publication bias.

## 4 Discussion

With more attention to the microbiota-gut-brain axis (MGBA), psychoprobiotics have been increasingly applied to the prevention and treatment of depression. Some experimental animal studies have found that germ-free mice transplanted with the microbiota of patients with major depression develop a typical depressive phenotype (45–47). Sudo et al. (48) found that infantile bifidobacterium colonization into germ-free mice weakened the hypothalamic-pituitary-adrenal axis (HPA) response and significantly improved depression-like behavior in mice.

As one of the most widely used psychoprobiotics, the mechanism of bifidobacterium in the treatment of depression involves multiple pathways, including the HPA axis, enteric nerve and immunoinflammatory response, central nervous system CNS, neurotransmitters, intestinal mucosa, and blood-brain barrier. One of the most consistent biological findings about the pathogenesis of depression is hyperactivity of the HPA axis. An animal experiment using Bifidobacterium breve to improve depressive symptoms in mice found that Bifidobacterium breve CCFM1025 could significantly alleviate HAP axis hyperactivity, inhibit hypothalamic corticotropin releasing factor (CRF) secretion, and reduce serum corticosterone levels (42).Hyperactivity of the HPA axis also leads to increased levels of inflammation in patients with depression (49).Jang et al. (50) found that Bifidobacterium longum NK98 can synergically alleviate depressive symptoms and the occurrence of colitis by regulating intestinal immune response and microbiota composition, producing effects similar to those of antidepressants; Yoo et al. (51)found that oral administration of Lactobacillus plantarum NK151, Bifidobacterium longum NK173, and bifidobacterium bifidum NK175 alleviated stress-induced depressive symptoms by regulating the expression ratio of pro-inflammatory and anti-inflammatory cytokines and intestinal microbiota byproducts (such as LPS).

In addition, the microbiota dominated by bifidobacterium mediates communication with the CNS using neurotransmitters such as gamma-aminobutyric acid (GABA), 5-hydroxytryptamine (5-HT), neuropeptides, corticotropin-releasing hormone secreted by the HPA axis, and short-chain fatty acids (such as ethylene hydrochloric acid) (52), For example, Bifidobacterium infantilum can affect central serotonin transmission by increasing plasma tryptophan (61), mediating MGBA bidirectional exchange to regulate metabolic and immune responses in patients since improving depression. The specific mechanism by which related intestinal probiotics affect the brain has not been fully clarified (53).

In this meta-analysis, we observed that the overall effect of Bifidobacterium on depressive symptoms was better than that of the placebo group. Three of the 13 studies included people with major depressive disorder (32, 36, 44);Four studies included people with mild to moderate depression (34, 35, 39, 40) and one study was depressed after stroke (59) and one study with chronic heart failure and depression (43), Four healthy volunteers with symptoms of subclinical depression (33, 37, 38, 41). The results of the study show that Bifidobacteria-related preparations have played a significant role in improving the depressive symptoms of patients with several types of depression. Sensitivity analyses showed no change in the conclusions of the meta-analysis when assessing differences between studies. Meta-analysis found that the incidence of depression was significantly reduced in the probiotic group, MD = -0.49(95%CI: -0.71, -0.26), p< 0. 0001. Individual studies did not report significant results.

Subgroup analysis showed that bifidobacteria-related preparations were significantly improved in patients with different degrees of depression., which was consistent with a previous meta-analysis (54). Probiotics were effective in reducing the depression rating scale in depressed patients and healthy volunteers with subclinical depressive symptoms. In addition, subgroup analyses showed that bifidobacteria and drugs were effective in reducing depression scores in participants. The effect was significantly better than that of patients treated with bifidobacterium alone, and we looked further at these studies in which the overall response rate was significantly higher in the experimental group with bifidobacteria than in the control group compared with the control group treated with antidepressant alone, which is consistent with the conclusions of Nikolova et al (55), indicating that Bifidobacteria-related preparations can be taken alone to reduce depressive symptoms, or as an adjuvant to the clinical treatment of depression, and combined with antidepressants to enhance the drug effect (56).

While we made efforts to conduct a thorough literature search to minimize publication bias in this meta-analysis, we did not exclude grey literature from our searches. Each step of the meta-analysis was performed by two independent investigators to reduce bias in the analysis, but there were some limitations to this study. First, it is worth noting that the study items used various depression rating scales, potentially impacting the results of the meta-analysis; Second, evidence suggests that the prevalence and onset of depression vary at different ages (57), neurobiological factors in adolescents and adults with depression vary widely (58), the abundance of bifidobacteria also varies in different age groups, (38, 40). The heterogeneity of the results of the two studies may be related to the large age span of the included patients. In the future, more relevant studies are needed to explore the improvement results of bifidobacterium on depression disorders in different age groups. As there are still relatively few literatures that can be screened, the subgroup analysis only simply divides depression types into “MDD” and “Others”, which may be one of the reasons for the large heterogeneity among studies. In the future, the sample size will be further increased to continue in-depth research on bifidobacterium treatment of depression.

## 5 Conclusions

This review has observed that bifidobacteria-related preparations are effective in the treatment of depression, and support the use of bifidobacteria-related preparations for the treatment of different symptoms of depression, as well as the reduction of symptoms of subclinical depression, and supports the future larger sample and more rigorous randomized controlled trials to conduct more in-depth research on the mechanism of bifidobacteria-related preparations in the treatment of depression.

## References

[1] Monroe SM Harkness KL. Major depression and its recurrences: life course matters. Annu Rev Clin Psychol. (2022) 18:329–57. doi: 10.1146/annurev-clinpsy-072220-021440 35216520

[2] Henssler J Heinz A Brandt L Bschor T. Antidepressant withdrawal and rebound phenomena. Dtsch Arztebl Int. (2019) 116:355–61. doi: 10.3238/arztebl.2019.0355 PMC6637660 31288917

[3] Dilsaver SC Greden JF. Antidepressant withdrawal phenomena. Biol Psychiatry. (1984) 19:237–56. 6324897

[4] Horowitz MA Framer A Hengartner MP Sørensen A Taylor D. Estimating risk of antidepressant withdrawal from a review of published data. CNS Drugs. (2023) 37:143–57. doi: 10.1007/s40263-022-00960-y PMC9911477 36513909

[5] Chudzik A Orzyłowska A Rola R Stanisz GJ. Probiotics, prebiotics and postbiotics on mitigation of depression symptoms: modulation of the brain-gut-microbiome axis. Biomolecules. (2021) 11(7):1000. doi: 10.3390/biom11071000 34356624 PMC8301955

[6] Liu RT Walsh RFL Sheehan AE. Prebiotics and probiotics for depression and anxiety: A systematic review and meta-analysis of controlled clinical trials. Neurosci Biobehav Rev. (2019) 102:13–23. doi: 10.1016/j.neubiorev.2019.03.023 31004628 PMC6584030

[7] Karakula-Juchnowicz H Rog J Juchnowicz D Łoniewski I Skonieczna-Żydecka K Krukow P. The study evaluating the effect of probiotic supplementation on the mental status, inflammation, and intestinal barrier in major depressive disorder patients using gluten-free or gluten-containing diet (SANGUT study): a 12-week, randomized, double-blind, and placebo-controlled clinical study protocol. Nutr J. (2019) 18:50. doi: 10.1186/s12937-019-0475-x 31472678 PMC6717641

[8] Schaub AC Schneider E Vazquez-Castellanos JF Schweinfurth N Kettelhack C Doll JPK. Clinical, gut microbial and neural effects of a probiotic add-on therapy in depressed patients: a randomized controlled trial. Transl Psychiatry. (2022) 12:227. doi: 10.1038/s41398-022-01977-z 35654766 PMC9163095

[9] Johnson D Thurairajasingam S Letchumanan V Chan KG Lee LH. Exploring the role and potential of probiotics in the field of mental health: major depressive disorder. Nutrients. (2021) 13(5):1728. doi: 10.3390/nu13051728 34065187 PMC8161395

[10] Okubo R Koga M Katsumata N Odamaki T Matsuyama S Oka M. Effect of bifidobacterium breve A-1 on anxiety and depressive symptoms in schizophrenia: A proof-of-concept study. J Affect Disord. (2019) 245:377–85. doi: 10.1016/j.jad.2018.11.011 30423465

[11] Tojo R Suárez A Clemente MG de los Reyes-Gavilán CG Margolles A Gueimonde M. Intestinal microbiota in health and disease: role of bifidobacteria in gut homeostasis. World J Gastroenterol. (2014) 20:15163–76. doi: 10.3748/wjg.v20.i41.15163 PMC4223251 25386066

[12] Johnson D Letchumanan V Thum CC Thurairajasingam S Lee LH. A microbial-based approach to mental health: the potential of probiotics in the treatment of depression. Nutrients. (2023) 15(6):1382. doi: 10.3390/nu15061382 36986112 PMC10053794

[13] Vlainić JV Šuran J Vlainić T Vukorep AL. Probiotics as an adjuvant therapy in major depressive disorder. Curr Neuropharmacol. (2016) 14:952–58. doi: 10.2174/1570159X14666160526120928 PMC5333591 27226112

[14] Xiong RG Li J Cheng J Zhou DD Wu SX Huang SY. The role of gut microbiota in anxiety, depression, and other mental disorders as well as the protective effects of dietary components. Nutrients. (2023) 15(14):3258. doi: 10.3390/nu15143258 37513676 PMC10384867

[15] Morales-Torres R Carrasco-Gubernatis C Grasso-Cladera A Cosmelli D Parada FJ Palacios-García I. Psychobiotic effects on anxiety are modulated by lifestyle behaviors: A randomized placebo-controlled trial on healthy adults. Nutrients. (2023) 15(7):1706. doi: 10.3390/nu15071706 37049546 PMC10096963

[16] Zommiti M Ferchichi M Feuilloley MGJ. "Beneficial microbes: food, mood and beyond"-editorial and the perspectives of research. Microorganisms. (2023) 11(4):1014. doi: 10.3390/microorganisms11041014 37110437 PMC10145506

[17] Rode J Edebol Carlman HMT König J Hutchinson AN Thunberg P Persson J. Multi-strain probiotic mixture affects brain morphology and resting state brain function in healthy subjects: an RCT. Cells. (2022) 11(18):2922. doi: 10.3390/cells11182922 36139496 PMC9496704

[18] Chahwan B Kwan S Isik A van Hemert S Burke C Roberts L. Gut feelings: A randomised, triple-blind, placebo-controlled trial of probiotics for depressive symptoms. J Affect Disord. (2019) 253:317–26. doi: 10.1016/j.jad.2019.04.097 31078831

[19] Bermúdez-Humarán LG Salinas E Ortiz GG Ramirez-Jirano LJ Morales JA Bitzer-Quintero OK. From probiotics to psychobiotics: live beneficial bacteria which act on the brain-gut axis. Nutrients. (2019) 11(4):890. doi: 10.3390/nu11040890 31010014 PMC6521058

[20] Socała K Doboszewska U Szopa A Serefko A Włodarczyk M Zielińska A. The role of microbiota-gut-brain axis in neuropsychiatric and neurological disorders. Pharmacol Res. (2021) 172:105840. doi: 10.1016/j.phrs.2021.105840 34450312

[21] Kim CS Cha L Sim M Jung S Chun WY Baik HW. Probiotic supplementation improves cognitive function and mood with changes in gut microbiota in community-dwelling older adults: A randomized, double-blind, placebo-controlled, multicenter trial. J Gerontol A Biol Sci Med Sci. (2021) 76:32–40. doi: 10.1093/gerona/glaa090 32300799 PMC7861012

[22] Zhang Q Chen B Zhang J Dong J Ma J Zhang Y. Effect of prebiotics, probiotics, synbiotics on depression: results from a meta-analysis. BMC Psychiatry. (2023) 23:477. doi: 10.1186/s12888-023-04963-x 37386630 PMC10308754

[23] Arboleya S Stanton C Ryan CA Dempsey E Ross PR. Bosom Buddies: The Symbiotic Relationship Between Infants and Bifidobacterium longum ssp. longum and ssp. infantis. Genetic and Probiotic Features. Annu Rev Food Sci Technol. (2016) 7:1–21. doi: 10.1146/annurev-food-041715-033151 26934170

[24] Hidalgo-Cantabrana C Delgado S Ruiz L Ruas-Madiedo P Sánchez B Margolles A. Bifidobacteria and their health-promoting effects. Microbiol Spectr. (2017) 5(3):10. doi: 10.1128/microbiolspec.BAD-0010-2016 PMC11687494 28643627

[25] Mills S Yang B Smith GJ Stanton C Ross RP. Efficacy of Bifidobacterium longum alone or in multi-strain probiotic formulations during early life and beyond. Gut Microbes. (2023) 15:2186098. doi: 10.1080/19490976.2023.2186098 36896934 PMC10012958

[26] Lai D Yang X Wu G Liu Y Nardini C. Inference of gene networks–application to Bifidobacterium. Bioinformatics. (2011) 27:232–7. doi: 10.1093/bioinformatics/btq629 21075742

[27] Wong CB Iwabuchi N Xiao JZ. Exploring the Science behind Bifidobacterium breve M-16V in Infant Health. Nutrients. (2019) 11(8):1724. doi: 10.3390/nu11081724 31349739 PMC6723912

[28] Turroni F van Sinderen D Ventura M. Genomics and ecological overview of the genus Bifidobacterium. Int J Food Microbiol. (2011) 149:37–44. doi: 10.1016/j.ijfoodmicro.2010.12.010 21276626

[29] Cheng J Laitila A Ouwehand AC. Bifidobacterium animalis subsp. lactis HN019 Effects on Gut Health: A Review. Front Nutr. (2021) 8:790561. doi: 10.3389/fnut.2021.790561 34970580 PMC8712437

[30] Chen M Xie CR Shi YZ Tang TC Zheng H. Gut microbiota and major depressive disorder: A bidirectional Mendelian randomization. J Affect Disord. (2022) 316:187–93. doi: 10.1016/j.jad.2022.08.012 35961601

[31] Altaib H Kozakai T Badr Y Nakao H El-Nouby MAM Yanase E. Cell factory for γ-aminobutyric acid (GABA) production using Bifidobacterium adolescentis. Microb Cell Fact. (2022) 21:33. doi: 10.1186/s12934-021-01729-6 35255900 PMC8903651

[32] Akkasheh G Kashani-Poor Z Tajabadi-Ebrahimi M Jafari P Akbari H Taghizadeh M. Clinical and metabolic response to probiotic administration in patients with major depressive disorder: A randomized, double-blind, placebo-controlled trial. Nutrition. (2016) 32:315–20. doi: 10.1016/j.nut.2015.09.003 26706022

[33] Boehme M Rémond-Derbez N Lerond C Lavalle L Keddani S Steinmann M. Bifidobacterium longum subsp. longum Reduces Perceived Psychological Stress in Healthy Adults: An Exploratory Clinical Trial. Nutrients. (2023) 15(14):3122. doi: 10.3390/nu15143122 37513541 PMC10383821

[34] Gawlik-Kotelnicka O Margulska A Skowrońska A Strzelecki D. PRO-DEMET randomized controlled trial on probiotics in depression-pilot study results. Nutrients. (2023) 15(6):1400. doi: 10.3390/nu15061400 36986132 PMC10058314

[35] Haghighat N Mohammadshahi M Shayanpour S Haghighizadeh MH Rahmdel S Rajaei M. The effect of synbiotic and probiotic supplementation on mental health parameters in patients undergoing hemodialysis: A double-blind, randomized, placebo-controlled trial. Indian J Nephrol. (2021) 31:149–56. doi: 10.4103/ijn.IJN_341_19 PMC8240938 34267437

[36] Kazemi A Noorbala AA Azam K Eskandari MH Djafarian K. Effect of probiotic and prebiotic vs placebo on psychological outcomes in patients with major depressive disorder: A randomized clinical trial. Clin Nutr. (2019) 38:522–28. doi: 10.1016/j.clnu.2018.04.010 29731182

[37] Lee HJ Hong JK Kim JK Kim DH Jang SW Han SW. Effects of probiotic NVP-1704 on mental health and sleep in healthy adults: an 8-week randomized, double-blind, placebo-controlled trial. Nutrients. (2021) 13(8):2660. doi: 10.3390/nu13082660 34444820 PMC8398773

[38] Patterson E Tan HTT Groeger D Andrews M Buckley M Murphy EF. Bifidobacterium longum 1714 improves sleep quality and aspects of well-being in healthy adults: a randomized, double-blind, placebo-controlled clinical trial. Sci Rep. (2024) 14:3725. doi: 10.1038/s41598-024-53810-w 38355674 PMC10866977

[39] Pinto-Sanchez MI Hall GB Ghajar K Nardelli A Bolino C Lau JT. Probiotic bifidobacterium longum NCC3001 reduces depression scores and alters brain activity: a pilot study in patients with irritable bowel syndrome. Gastroenterology. (2017) 153:448–59.e8. doi: 10.1053/j.gastro.2017.05.003 28483500

[40] Romijn AR Rucklidge JJ Kuijer RG Frampton C. A double-blind, randomized, placebo-controlled trial of Lactobacillus helveticus and Bifidobacterium longum for the symptoms of depression. Aust N Z J Psychiatry. (2017) 51:810–21. doi: 10.1177/0004867416686694 PMC5518919 28068788

[41] Steenbergen L Sellaro R van Hemert S Bosch JA Colzato LS. A randomized controlled trial to test the effect of multispecies probiotics on cognitive reactivity to sad mood. Brain Behav Immun. (2015) 48:258–64. doi: 10.1016/j.bbi.2015.04.003 25862297

[42] Tian P Wang G Zhao J Zhang H Chen W. Bifidobacterium with the role of 5-hydroxytryptophan synthesis regulation alleviates the symptom of depression and related microbiota dysbiosis. J Nutr Biochem. (2019) 66:43–51. doi: 10.1016/j.jnutbio.2019.01.007 30743155

[43] Yao D. Clinical observation of Qili Qiangxin Capsule combined with Bifidobacterium quadruple viable tablets in the adjuvant treatment of chronic heart failure complicated with depression. J Pract Chin Med. (2023) 39:725–7.

[44] Tian P Chen Y Zhu H Wang L Qian X Zou R. Bifidobacterium breve CCFM1025 attenuates major depression disorder via regulating gut microbiome and tryptophan metabolism: A randomized clinical trial. Brain Behav Immun. (2022) 100:233–41. doi: 10.1016/j.bbi.2021.11.023 34875345

[45] Jang HM Kim JK Joo MK Shin YJ Lee CK Kim HJ. Transplantation of fecal microbiota from patients with inflammatory bowel disease and depression alters immune response and behavior in recipient mice. Sci Rep. (2021) 11:20406. doi: 10.1038/s41598-021-00088-x 34650107 PMC8516877

[46] Hao W Ma Q Wang L Yuan N Gan H He L. Gut dysbiosis induces the development of depression-like behavior through abnormal synapse pruning in microglia-mediated by complement C3. Microbiome. (2024) 12:34. doi: 10.1186/s40168-024-01756-6 38378622 PMC10877840

[47] Zheng P Zeng B Zhou C Liu M Fang Z Xu X. Gut microbiome remodeling induces depressive-like behaviors through a pathway mediated by the host's metabolism. Mol Psychiatry. (2016) 21:786–96. doi: 10.1038/mp.2016.44 27067014

[48] Sudo N Aiba Y Oyama N Yu XN Matsunaga M Koga Y. Dietary nucleic acid and intestinal microbiota synergistically promote a shift in the Th1/Th2 balance toward Th1-skewed immunity. Int Arch Allergy Immunol. (2004) 135:132–5. doi: 10.1159/000080655 15345911

[49] Wong ML Inserra A Lewis MD Mastronardi CA Leong L Choo J. Inflammasome signaling affects anxiety- and depressive-like behavior and gut microbiome composition. Mol Psychiatry. (2016) 21:797–805. doi: 10.1038/mp.2016.46 27090302 PMC4879188

[50] Jang HM Lee KE Kim DH. The Preventive and Curative Effects of Lactobacillus reuteri NK33 and Bifidobacterium adolescentis NK98 on Immobilization Stress-Induced Anxiety/Depression and Colitis in Mice. Nutrients. (2019) 11(4):819. doi: 10.3390/nu11040819 30979031 PMC6521032

[51] Yoo JW Shin YJ Ma X Son YH Jang HM Lee CK. The alleviation of gut microbiota-induced depression and colitis in mice by anti-inflammatory probiotics NK151, NK173, and NK175. Nutrients. (2022) 14(10):2080. doi: 10.3390/nu14102080 35631220 PMC9147079

[52] Gan Y Chen Y Zhong H Liu Z Geng J Wang H. Gut microbes in central nervous system development and related disorders. Front Immunol. (2023) 14:1288256. doi: 10.3389/fimmu.2023.1288256 38343438 PMC10854220

[53] Sorboni SG Moghaddam HS Jafarzadeh-Esfehani R Soleimanpour S. A comprehensive review on the role of the gut microbiome in human neurological disorders. Clin Microbiol Rev. (2022) 35:e0033820. doi: 10.1128/CMR.00338-20 34985325 PMC8729913

[54] Huang R Wang K Hu J. Effect of probiotics on depression: A systematic review and meta-analysis of randomized controlled trials. Nutrients. (2016) 8(8):483. doi: 10.3390/nu8080483 27509521 PMC4997396

[55] Nikolova VL Cleare AJ Young AH Stone JM. Acceptability, tolerability, and estimates of putative treatment effects of probiotics as adjunctive treatment in patients with depression: A randomized clinical trial. JAMA Psychiatry. (2023) 80:842–47. doi: 10.1001/jamapsychiatry.2023.1817 PMC10267847 37314797

[56] Musazadeh V Zarezadeh M Faghfouri AH Keramati M Jamilian P Jamilian P. Probiotics as an effective therapeutic approach in alleviating depression symptoms: an umbrella meta-analysis. Crit Rev Food Sci Nutr. (2023) 63:8292–300. doi: 10.1080/10408398.2022.2051164 35348020

[57] Wagner S Wollschläger D Dreimüller N Engelmann J Herzog DP Roll SC. Effects of age on depressive symptomatology and response to antidepressant treatment in patients with major depressive disorder aged 18 to 65 years. Compr Psychiatry. (2020) 99:152170. doi: 10.1016/j.comppsych.2020.152170 32146314

[58] Kaufman J Martin A King RA Charney D. Are child-, adolescent-, and adult-onset depression one and the same disorder?. Biol Psychiatry. (2001) 49:980–1001. doi: 10.1016/S0006-3223(01)01127-1 11430841

[59] Chen Y Peng B Yan H Su Y. Clinical study of Bifidobacterium quadruple viable bacteria combined with fluoxetine in patients with post-stroke depression. Electronic J Clin Med Literature. (2022) 9:5–8.

[60] WHO. (2023). Available online at: https://www.who.int/news-room/fact-sheets/detail/depression.

[61] Luk B Veeraragavan S Engevik M Balderas M Major A Runge J. Postnatal colonization with human “infant-type” Bifidobacterium species alters behavior of adult gnotobiotic mice. PLoS One. (2018) 13(5):e0196510. doi: 10.1371/journal.pone.0196510 29763437 PMC5953436

